# AiroTouch: enhancing telerobotic assembly through naturalistic haptic feedback of tool vibrations

**DOI:** 10.3389/frobt.2024.1355205

**Published:** 2024-05-21

**Authors:** Yijie Gong, Haliza Mat Husin, Ecda Erol, Valerio Ortenzi, Katherine J. Kuchenbecker

**Affiliations:** ^1^ Haptic Intelligence Department, Max Planck Institute for Intelligent Systems, Stuttgart, Germany; ^2^ Mechanical Engineering, University of Stuttgart, Stuttgart, Germany

**Keywords:** teleoperation, assembly tasks, vibrotactile feedback, audio equipment, user experience

## Abstract

Teleoperation allows workers to safely control powerful construction machines; however, its primary reliance on visual feedback limits the operator’s efficiency in situations with stiff contact or poor visibility, hindering its use for assembly of pre-fabricated building components. Reliable, economical, and easy-to-implement haptic feedback could fill this perception gap and facilitate the broader use of robots in construction and other application areas. Thus, we adapted widely available commercial audio equipment to create AiroTouch, a naturalistic haptic feedback system that measures the vibration experienced by each robot tool and enables the operator to feel a scaled version of this vibration in real time. Accurate haptic transmission was achieved by optimizing the positions of the system’s off-the-shelf accelerometers and voice-coil actuators. A study was conducted to evaluate how adding this naturalistic type of vibrotactile feedback affects the operator during telerobotic assembly. Thirty participants used a bimanual dexterous teleoperation system (Intuitive da Vinci Si) to build a small rigid structure under three randomly ordered haptic feedback conditions: no vibrations, one-axis vibrations, and summed three-axis vibrations. The results show that users took advantage of both tested versions of the naturalistic haptic feedback after gaining some experience with the task, causing significantly lower vibrations and forces in the second trial. Subjective responses indicate that haptic feedback increased the realism of the interaction and reduced the perceived task duration, task difficulty, and fatigue. As hypothesized, higher haptic feedback gains were chosen by users with larger hands and for the smaller sensed vibrations in the one-axis condition. These results elucidate important details for effective implementation of naturalistic vibrotactile feedback and demonstrate that our accessible audio-based approach could enhance user performance and experience during telerobotic assembly in construction and other application domains.

## 1 Introduction

Teleoperation has been shown to increase safety and comfort for human workers in applications such as minimally invasive surgery, search and rescue, and construction (Niemeyer et al., 2016). In telerobotic construction activities, the operator usually controls the robot using joysticks and levers, standing at a distance from the end-effector to ensure their own physical safety (Saidi et al., 2016). In such scenarios, operators rely predominantly on direct vision or camera feeds to operate the robot. Given the chaotic outdoor setting, this visual feedback often suffers from poor viewing conditions and complete occlusion, leading to uncontrolled interactions between the robot and its environment, especially during the contact-heavy process of assembly (Melenbrink et al., 2020). Typically, a construction robot with an end-effector securely grips the chosen building component, lifts it up slowly, and relocates it near the desired assembly position. Then, the operator adjusts the component’s position and orientation to carefully achieve its final placement relative to the stiff existing structure (Gong et al., 2023a). This final step has low tolerance for error, so the operator often needs multiple attempts to find the optimal pose (Gong et al., 2023a), which increases the risk of damage to the manipulated component and the building. Researchers have thus been working on augmenting the operator’s capabilities by equipping robots with sensors (e.g., Sun et al., 2017) and providing operators with haptic feedback (e.g., Culbertson et al., 2018) to reduce their workload.

Haptic feedback systems in teleoperation usually measure or estimate the contact force of the robot and display that force to the user in real time (Niemeyer et al., 2016; Kuchenbecker, 2018), thereby providing *direct force feedback*. Horie et al. (2001) and Hirabayashi et al. (2006) used force sensors to measure the force applied to the robot end-effector and displayed corresponding forces through a PHANToM haptic device and a customized device, respectively. Similarly, Tang et al. (2009) developed a haptic feedback system for an excavator to provide the operator with estimated force feedback via a motor-driven joystick; their user study showed that this type of force feedback decreased both the applied force and the contact time in a block movement task. As a drawback, most of these grounded force-feedback systems are designed to be affixed to a table with a monitor, which is impractical for assembling buildings, as the operator needs to move around the construction site. Additionally, three-axis force sensors are typically too bulky, delicate, and expensive to be included in construction machines.

Researchers have also explored haptic sensory substitution, i.e., displaying telerobotic contact signals through other feedback channels. For instance, early work by Massimino and Sheridan (1992) mapped the magnitude of the measured force to the peak-to-peak magnitude of a fixed-frequency vibration that the user could feel. Their user study showed high success rate and time efficiency in peg-in-hole tasks with this type of vibration feedback, though the presented haptic sensations differ from those felt in natural physical interactions. In a recent application of haptic sensory substitution in construction, Nagano et al. (2020) measured the high-frequency acceleration of the robot manipulator with a one-axis piezoelectric sensor and presented this information via an amplitude-modulated fixed-frequency vibration using a voice-coil actuator on the user’s wrist. The associated user study showed that this type of feedback decreased the peak force in a bar insertion task.

*Naturalistic vibrotactile feedback* provides an appealing alternative to direct force feedback and haptic sensory substitution (Kuchenbecker et al., 2010; McMahan et al., 2011). We define that a system that measures real vibration signals from a physical source and replays them to the user without manipulating the signal’s spectrogram across a broad band of frequencies can be considered as providing naturalistic vibrotactile feedback, since the vibrations felt match the natural vibrations occurring at the robot’s end-effector. Generally, vibrations are simpler to measure and output than forces in an unstructured environment, as vibrotactile sensors and actuators are usually small and inexpensive, and they do not restrict operator movement. Moreover, human vibrotactile perception has a wide bandwidth up to 1,000 Hz (Bell et al., 1994), which includes the high frequencies that are typically stimulated in contacts between rigid objects being assembled. This capability has inspired researchers to provide vivid broad-bandwidth haptic feedback during teleoperation. In a first effort, Kontarinis and Howe (1995) designed a two-fingered teleoperation system with both direct force feedback and naturalistic vibrotactile feedback; one axis of the vibrations on the robot’s fingertip was measured by an accelerometer and played back to the user via an inverted loudspeaker. Their study showed that naturalistic vibrotactile feedback helps reduce the user’s reaction time and the force exerted during a puncture task.

More recently, VerroTouch (Kuchenbecker et al., 2010; McMahan et al., 2011) was developed as a naturalistic vibrotactile feedback system for da Vinci robots (Intuitive Surgical, Inc.), which natively have no haptic feedback. VerroTouch uses three-axis MEMS-based accelerometers to measure the left and right robotic tool vibrations and custom voice-coil actuators attached to the da Vinci handles to display the vibrations to the user’s hands. The initial version used a single accelerometer axis (Kuchenbecker et al., 2010), and the later version summed all three axes (McMahan et al., 2011), but this difference was never investigated. Studies with surgeons and non-surgeons showed strong preferences for having this type of vibrotactile feedback in robotic surgery training tasks because it strengthens the operator’s connection to the tools (McMahan et al., 2011; Koehn and Kuchenbecker, 2014), but no quantitative differences in task performance were ever demonstrated, potentially due to insufficient exposure time. VerroTouch was created with custom electronics for use on da Vinci robots, but its approach can be adapted to other teleoperated robots and operator interfaces, including handheld devices with no connection to ground (Khurshid et al., 2017; Kuchenbecker et al., 2017), as might be used on a construction site. Takahashi et al. (2023) recently adopted a similar approach for teleoperated material discrimination with a grounded haptic interface. Their system uses a piezoelectric accelerometer to measure the one-axis vibrations of a robot tool and an audio interface to transmit these vibration signals to a voice-coil transducer on the operator’s palm; adding naturalistic vibrotactile feedback to force feedback was found to improve roughness discrimination but not hardness discrimination (Takahashi et al., 2023). Compared with vibrations that represent forces through sensory substitution, broad-bandwidth vibrations are more intuitive because they reproduce natural physical contact sensations that humans feel when directly using tools.

**FIGURE 1:**
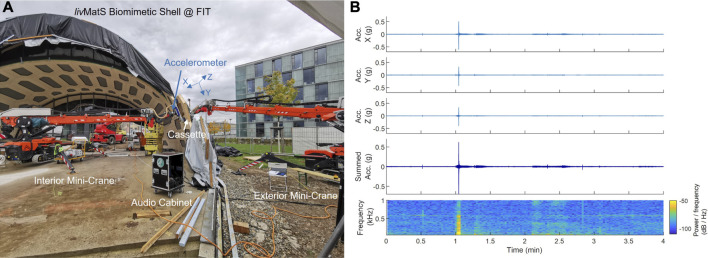
**(A)** AiroTouch, our naturalistic vibrotactile feedback system, installed on a construction site. The two mini-crane robots worked together to assemble a timber pavilion (*liv*MatS Biomimetic Shell @ FIT). **(B)** Acceleration signals measured during a sample assembly process: The three orthogonal vibration signals were measured by the accelerometer on the interior mini-crane, and the summed vibration was experienced by the observing participant if haptic feedback was being provided. The acceleration signal’s spectrogram shows the instantaneous frequency content of the summed vibration. Each transient peak indicates a new contact between the robot end-effector and either the wooden cassette or the structure.

In this context, we designed a reliable, economical, and easy-to-implement system for providing naturalistic vibrotactile feedback during teleoperation: AiroTouch uses widely available off-the shelf audio and haptic components to enable the user to feel a scaled version of the broad-bandwidth vibration that a robot’s end-effector is experiencing in real time. Section 2 explains the system’s working mechanism, which was preliminarily introduced in a conference paper by Gong et al. (2023a), and discusses how to optimize its application to a particular teleoperation system. Importantly, this first investigation of AiroTouch also reported a study wherein naive users felt its feedback while observing (but not controlling) the assembly of pre-fabricated wooden building components on a real construction site. Figure 1 shows the two mini-crane robots that performed the assembly (Lauer et al., 2023), along with sample vibration signals from the interior robot (Gong et al., 2023b). The user ratings and comments revealed that naturalistic vibrotactile feedback improved the participant’s awareness of contacts, and they believed that this type of feedback would be beneficial for telerobotic assembly tasks in construction (Gong et al., 2023a). However, safety and liability concerns have thus far prohibited testing of our haptic feedback approach during teleoperation of a real construction robot to perform assembly tasks; specifically, we needed to avoid potential disruption to ongoing construction activities, the risk of injuries or accidents that could harm the structure or equipment, and potential economic loss due to the experiment. Simulation is also not suitable because it would require an accurate model for the generation of contact vibrations. Thus, this article builds on the initial validation of AiroTouch for construction (Gong et al., 2023a) by evaluating how this type of haptic feedback affects a person remotely controlling a similar robot to perform a similar assembly task.

We are curious about how to implement a naturalistic vibrotactile feedback system to achieve good performance on a particular robotic system; specifically, where should the sensors and actuators be placed, and can users distinguish between one-axis and summed three-axis feedback? Furthermore, we want to quantify the objective and subjective effects that this kind of feedback has on users during telerobotic assembly. We hypothesize that naturalistic vibrotactile feedback of tool vibrations will:

***H1:*** differ in quality depending on the locations of the sensors and actuators,

***H2:*** help the user create smaller tool vibrations and exert smaller forces on the structure being assembled,

***H3:*** increase the user’s confidence and decrease their mental stress, and

***H4:*** be enhanced by displaying the sum of all three orthogonal vibrations rather than a single axis.

Past work showed that users exhibit varying preferences for the magnitude of naturalistic vibrotactile feedback (McMahan et al., 2011; Koehn and Kuchenbecker, 2014), which might be influenced by the intensity of the measured vibration signal as well as user characteristics such as age, gender, and tactile sensitivity. Dynamic modeling predicts that when the user has a larger hand, the force required to vibrate this increased mass needs be stronger to generate vibrations of the same magnitude (McMahan and Kuchenbecker, 2014). Thus, we hypothesize that:

***H5:*** in general, a user’s preferred feedback gain positively correlates with their hand size and negatively correlates with the intensity of the measured vibration signal.

Given the challenge of accessing a construction site and the importance of operator safety, we decided to conduct preliminary testing before deploying our system for teleoperation of a construction robot. In this paper, we test our hypotheses with a commercial teleoperation system on a smaller scale. We use an Intuitive da Vinci Si robot, as it accurately maps human motion to robot motion and provides no form of haptic feedback. Although the length scales are different, assembling building components with mini-crane construction robots and minimally invasive surgical robots requires the same operations of picking up objects, moving them into position, and gently assembling them at the target locations. Furthermore, both robots have long, thin arms that pivot around a fixed point and extend to reach the items being manipulated. Indeed, the acceleration plots in Figure 1 reveal that the patterns of the vibration signals during construction robot assembly closely resemble those previously recorded with VerroTouch on da Vinci systems (Kuchenbecker et al., 2010; McMahan et al., 2011), giving us confidence that such a robot is suitable for this experimental evaluation of AiroTouch. Section 3 describes the methods of our user study. The results are presented in Section 4 and discussed in Section 5. We conclude with implications, limitations, and future potentials of our work in Section 6.

## 2 Haptic feedback system

**FIGURE 2:**
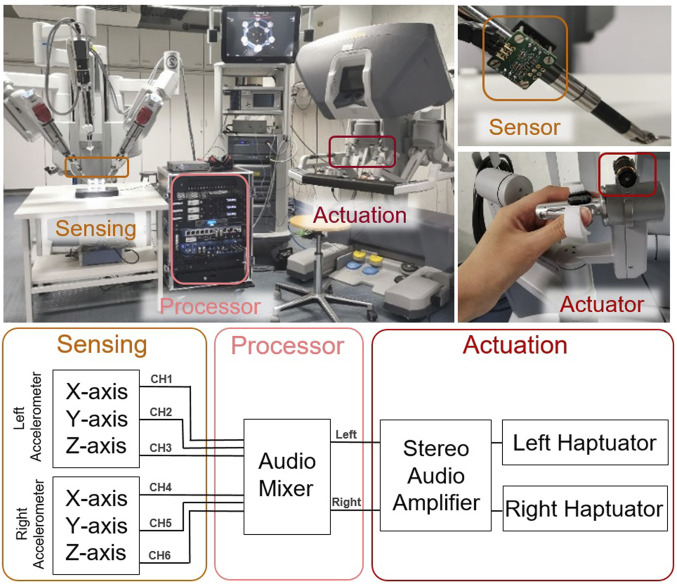
AiroTouch installed on a da Vinci robot. It comprises two accelerometers for vibration sensing, off-the-shelf audio equipment for processing (an audio mixer) and amplification (a stereo audio amplifier), and two voice-coil actuators to output the vibrations for the user to feel in real time.

Our audio-based system for providing naturalistic vibrotactile feedback consists of sensing, processing, and actuation units, as shown in Figure 2 on a da Vinci Si robot. The design of AiroTouch was inspired by several other haptic systems that employed audio technology, such as recording VerroTouch vibrations as audio signals (McMahan et al., 2013), adapting audio processing techniques to compress vibrotactile signals (Chaudhari et al., 2012), outputting vibrotactile signals with a sound card and an audio amplifier (Pezent et al., 2021), and generating haptic vibrations with an audio speaker (Minamizawa et al., 2012; Israr et al., 2019). AiroTouch includes two three-axis high-bandwidth accelerometers attached on the two tools of the da Vinci to measure vibrations, an audio mixer that processes the sensed vibrations and outputs them to the actuation unit, and two commercial voice-coil actuators that receive the amplified signals from a stereo audio amplifier and play the sensed vibrations to the user.

### 2.1 System design

Human vibrotactile perception provides a baseline for the minimal bandwidth requirement: 20–1,000 Hz (Bell et al., 1994). Thus, we chose components that all have suitable frequency bandwidth to guarantee high-fidelity transmission of the vibration experienced by each tool. For the sensing unit, two three-axis accelerometers on evaluation boards (Analog Devices EVAL-ADXL 356b, 20 mm × 20 mm × 5 mm, 0–1,500 Hz bandwidth) were selected and attached to the two tools of the robot to capture their vibrations. For the processor unit, a digital audio mixer (Soundcraft Ui24R) with a band-pass filter set to 80–1,000 Hz mixes, filters, and adjusts the magnitude of the signals from the accelerometers. This frequency range is used to filter out 50 Hz electrical noise, suppress the low-frequency ego-vibrations of the da Vinci robot, and prevent sustained vibrations that can be caused by closed-loop instability (McMahan et al., 2011). Note that the bandwidth of the filter may need to be adjusted for different robots.

For the actuation unit, a stereo audio amplifier (Renkforce T21, 20–20 000 Hz) with a maximum output power of 50 dB per channel drives both actuators (Tactile Labs Haptuator Redesign). Each of the two employed Haptuators (16 mm diameter × 29 mm, > 1,000 Hz bandwidth) provides high-quality one-axis haptic vibration feedback to one of the operator’s hands (McMahan and Kuchenbecker, 2014). The routing of accelerometer axes to output channels can be easily reconfigured in the mixer’s graphical user interface, and the strength of the haptic feedback is adjusted through the master volume control. All components in our telerobotic system (the da Vinci Si robot and the haptic feedback system) share the same electrical ground to avoid systematic noise.

The total cost of the AiroTouch components reported here amounts to approximately 1200 USD. This same haptic feedback system can easily be attached to other teleoperated robots by modifying the mounting brackets for the sensors and actuators. Gong et al. (2023a) report adaptation of AiroTouch for a Jekko mini-crane SPX532, using one three-axis accelerometer, three wireless transmitter/receiver pairs for audio input, one audio mixer, one wireless transmitter/receiver pair for audio output, one audio amplifier, and one Haptuator held directly in the user’s hand; such a configuration of AiroTouch costs about 2600 USD.

***H1*** hypothesizes that the placement and orientation of AiroTouch’s components will influence the quality of the naturalistic vibrotactile feedback it can provide on a particular robot. Section 2.2 investigates how the accelerometer’s sensitivity to contacts on the tool end-effector depends on its mounting location. Similarly, Section 2.3 discusses how the Haptuator’s ability to accurately vibrate the user’s fingers at a broad range of frequencies depends on how it is mounted.

### 2.2 Positioning the accelerometer on the tool

To systematically select good mounting locations for the accelerometers and thus provide the user with realistic feedback of the tool contact vibrations, we conducted tests at several potential accelerometer locations. We intended to increase the measured strength of vibrations originating from contact at the robot end-effector and simultaneously reduce the measured strength of internal robot vibrations caused by tool translation and rotation. Regardless of their source, induced vibrations travel along stiff structures with gradually attenuated strengths and are strongly affected by structural resonances.

**FIGURE 3:**
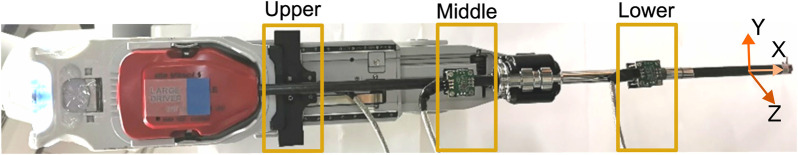
Three possible locations for mounting the accelerometer.

Custom 3D-printed brackets were used to mount identical accelerometers at three different locations on one tool of the robot, as shown in Figure 3. The upper location is next to the mounting point of the robot tool, the same location as that of VerroTouch (McMahan et al., 2011). The middle location is on the tool shaft (Brown et al., 2017), and the lower location is on the cannula, a steel tube that supports the tool. The vibration during three teleoperated actions was captured for about 15 s each: 1) rotation, where the tool rotates around its central *x*-axis; 2) motion, including translation and rotation, where the tool moves in all directions to simulate the reaching process; and 3) contact, where the end-effector collides with another object. We chose these basic actions as they often occur in assembly tasks, for example, when moving and rotating the tool to relocate an object. Vibration measurements were also collected for 15 s while the user was idle to capture the background noise of the robot and sensing system.

The ratio between the acceleration signal energy (ASE) of each action and the idle signal energy was used to evaluate the signal quality at each sensor location. The ASE for a single axis is calculated as:

$$E_{i}=\int_{-\infty }^{\infty }|a_{i}\left(t\right){|}^{2}dt$$ (1)

where *a*_*i*_ is the acceleration recorded over time on axis *i*. The ratio (*ρ*) between the total ASE of each action (*α*) and the total ASE of the idle state (∗) was calculated as:

$$\rho^{\alpha }=\frac{E_{x}^{\alpha }+E_{y}^{\alpha }+E_{z}^{\alpha }}{E_{x}^{*}+E_{y}^{*}+E_{z}^{*}}$$ (2)

A higher energy ratio means the corresponding location captures more vibration information for that action. To emphasize physical contacts over the robot’s own movements, a low ratio is desirable for rotation and motion, while a high ratio is desirable for contact. As shown in Table 1, the accelerometers at the middle and lower locations have a higher energy ratio during contact. However, the accelerometer at the middle location measured strong distracting vibrations during rotation and thus had the highest energy ratio for that action; these vibrations seem to originate from the internal bearing supporting the tool shaft. Therefore, the lower location is the best option for accelerometer placement in this study.

**TABLE 1:** For each mounting position shown in Figure 3, we computed the ratios between the total acceleration signal energy of each action (rotation, motion, and contact) and that of the idle state. The rotation and motion actions are marked with *↓* because smaller ratios indicate better performance. In contrast, the contact action is marked with *↑* because better performance is indicated by larger ratios.

Action	Upper position	Middle position	Lower position
Rotation *↓*	2.53 ± 1.42	157.15 ± 160.61	10.98 ± 6.30
Motion *↓*	4.70 ± 0.26	10.87 ± 0.05	8.93 ± 1.39
Contact *↑*	200.31 ± 107.85	2,409.51 ± 1,208.45	4,943.08 ± 3,012.26

To better understand how tool contact causes the tested robot to vibrate at each accelerometer location, we also analyzed the energy ratio for each axis during contact. As shown in Table 2, the ratios of the three axes are similar for the upper and middle locations, while the *x*-axis of the lower location performs worse than the other two axes. These differences likely stem from the structure of the tool. These findings support the first half of ***H1***: The quality of the sensed vibration depends on where the accelerometer is mounted.

**TABLE 2:** For each combination of accelerometer axis and mounting position, we computed the energy ratio for the contact action compared to the idle state. A larger ratio indicates that a configuration experiences stronger contact acceleration signals.

Acc. Axis	Upper position	Middle position	Lower position
X	30.20 ± 20.23	384.12 ± 178.49	748.55 ± 507.14
Y	269.14 ± 104.78	3,142.24 ± 1766.56	7,612.03 ± 4,793.68
Z	328.44 ± 208.97	3,737.57 ± 1,635.37	6,307.45 ± 3,595.00

### 2.3 Positioning the actuator on the handle

The quality of the vibrotactile feedback is limited by the actuator’s capacity to replay the tool-contact vibrations on the handle. To provide naturalistic vibrations, distortion and attenuation from the actuators to the hands should be minimal.

**FIGURE 4:**
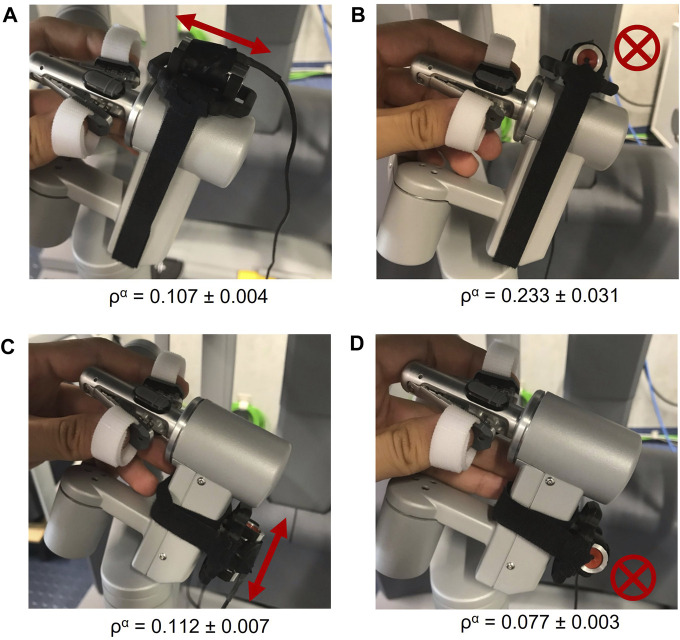
Tested locations of the actuator on the left handle: **(A)** parallel to the upper part of the handle, **(B)** perpendicular to the upper part of the handle, **(C)** parallel to the lower part of the handle, and **(D)** perpendicular to the lower part of the handle. The arrows and crosses show the direction of the actuator’s vibration output in each location. The energy ratio (*ρ*) results are shown below each location as mean ± standard deviation.

We considered four possible locations for the actuator on the operator’s handle, as shown in Figure 4. To compare the quality of the vibration signals, we fed the actuator with the same contact signals. A three-axis accelerometer was rigidly attached close to where the user’s fingertips hold the handle to record the actual vibration feedback. The energy ratio between the total ASE of the vibration generated by the actuator on the handle (*α*) and the acceleration source (∗) was calculated using Eq. 1 and Eq. 2. A higher ratio means better vibrotactile signal transmission to the user’s fingers. As shown in Figure 4, the ratio is clearly highest when the actuator is mounted in position (b). Therefore, we decided to use this location for our study. These findings support the second half of ***H1***: The quality of the actuated vibration depends on where the voice coil is mounted.

### 2.4 Signal reproduction from the tool to the handle

We then measured the quality of the reproduced signals to ensure that the user feels a naturalistic reproduction of the vibration occurring at each tool. We measured this quality indicator using four identical accelerometers, which we attached on the two robot tools in the selected lower location and on the handles near the selected location for the actuators. An experimenter teleoperated the robot for the three actions mentioned in Section 2.2: rotation, motion, and contact. The signals from the accelerometers attached to the tools were processed by the audio mixer, and the accelerometers on the handles measured the vibrations from the actuators. Since humans cannot easily perceive the direction of vibrations (Hwang et al., 2013), we convert the three-axis acceleration signals to a one-axis signal by simple arithmetic summing in the time domain, as done in some prior work (Landin et al., 2010; McMahan et al., 2011). This three-to-one function is easily configured in our audio mixer, and perceptual studies (Park and Kuchenbecker, 2019; Lee et al., 2022) demonstrated its efficacy at informatively presenting the original three-axis vibration signals.

As articulated in ***H4***, we hypothesize that these summed acceleration signals have more information than any of the individual one-axis signals from the accelerometer, since contact vibrations can occur in all directions; we test this hypothesis in Section 3. The similarity of the summed signals measured on the robot tools and the robot handles during the quality experiment was analyzed using cross-correlation (xcorr and corrcoef in MATLAB). The results show that the accelerations on each handle are similar to the signal from the corresponding accelerometer (*R*_left_ = 0.454, *R*_right_ = 0.463, *p* < 0.001 and *t*_delay_ = 0.042 s for both), proving that AiroTouch reproduces the vibrotactile signal accurately and with minimal time delay.

Compared with VerroTouch (McMahan et al., 2011), the presented naturalistic vibrotactile feedback system is easy to build from off-the-shelf components, requires no custom electronics, and allows quick reconfiguration through the mixer. Furthermore, it provides real-time vibrotactile feedback with lower noise and less signal attenuation than VerroTouch due to superior sensing, processing, and actuation. Therefore, this accessible technical approach is capable of providing high-fidelity haptic feedback for telerobotic construction and many other applications.

## 3 Experimental evaluation

We conducted a user study to evaluate how the naturalistic vibrotactile feedback provided by AiroTouch affects operator performance during telerobotic tasks inspired by on-site assembly of pre-fabricated building components.

### 3.1 Participants

Thirty adults participated in the study (13 female, 17 male; age ∈ [23, 40], 28.6 (mean) ± 3.49 (standard deviation)). They were recruited from a local university population, and twenty-six of them had an engineering background. When asked on a scale from 0 (novice) to 100 (expert), most participants had significant experience with assembly tasks such as putting together LEGO bricks or IKEA furniture (68.5 ± 28.3). Few were familiar with telerobotics (11.7 ± 14.6) or haptics research (11.9 ± 16.0). All participants were healthy and right-handed, and all reported normal or corrected-to-normal vision. All gave written informed consent, and people who did not work for our institution were paid 8 euros per hour for participation. The experiment followed procedures approved under the Haptic Intelligence framework agreement from the Max Planck Ethics Council with protocol number F012B.

### 3.2 Experimental procedure

**FIGURE 5:**
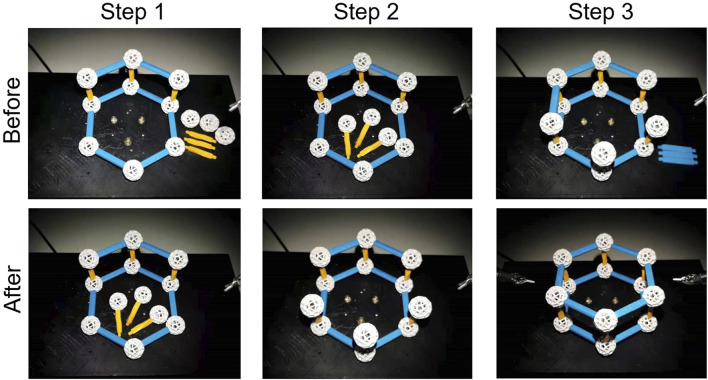
In step 1 of the construction task, the participant assembles three pairs of yellow sticks and white spheres. In step 2, they insert the assembled parts into three base spheres. In step 3, they connect the upper spheres with four blue sticks to form the final structure.

We asked participants to perform a three-step assembly task. For each trial, they needed to assemble construction toys (Zometool Inc.) from a half-built state (Figure 5, upper left) to a fully finished state (Figure 5, lower right). The construction toys comprise sticks and hollow spheres; the assembly procedure includes picking up the parts, moving and inserting parts into other parts, and attaching them to the existing structure. The detailed procedure has the following three steps:

Step 1: connect three pairs of yellow sticks and spheres;

Step 2: insert the yellow sticks into three base spheres;

Step 3: connect the upper spheres with four blue sticks.

Participants viewed the task materials through the da Vinci Si’s stereoscopic endoscope, and they manipulated the task materials with two needle drivers at normal motion scaling. The setup of the camera and tools was standardized, and participants were not allowed to move the camera or clutch the tools.

To test ***H2***, we recorded the vibrations of both tools and used a six-axis force-torque sensor (ATI Mini40) under the construction baseplate to record the forces applied to the task materials; it was zeroed before each trial. To help evaluate our haptic system’s effects on mental stress (***H3***), participants wore a wrist-mounted tracker (Polar OH1) to collect their heart rate during the experiment. Participants also wore passive noise-canceling headphones to mask ambient sounds.

The study included three different vibrotactile feedback conditions to investigate the impact of our haptic system: without feedback (F0), with the feedback from only one accelerometer axis (F1), and with the feedback computed from summing all three axes of the respective accelerometer (F3). The F1 and F3 conditions were designed to test our hypothesis ***H4*** that summed three-axis vibrotactile feedback is more informative than one axis. F1 used the accelerometer’s *x*-axis, which aligns with the tool shaft, as it is less sensitive to the direction of contact than the two other axes.

To evaluate ***H5*** about hand size, we measured each participant’s hand volume using a container filled with water on top of a digital scale (Hughes and Lau, 2008). Each hand of the participant was measured twice up to the wrist joint, and the mean volume for each participant was recorded.

Two questionnaires were administered to collect subjective feedback, as shown in Table 3. The post-trial questionnaire was used to record the participant’s subjective impression immediately after each trial. It consists of four customized questions (Q1–Q3, Q5) and six questions about workload, directly adapted from the NASA task load index (TLX, Q4.1–Q4.6): They focus on mental demand, physical demand, temporal demand, performance, effort, and frustration (Hart and Staveland, 1988). Each question has a scale from 0 (not at all) to 100 (completely). The post-experiment questionnaire was designed to compare performance across trials. It includes three free-response questions (Q6, Q11, Q12) and four questions regarding preference among the three trials (Q7–Q10). Participants saw an overview of these questionnaires before the corresponding trials.

**TABLE 3:** Questionnaires completed during the study.

	Post-trial questions
Q1	How confident were you in moving the building materials? (0–100)
Q2	How confident were you in assembling the structure? (0–100)
Q3	How realistic was your interaction with the building materials? (0–100)
Q4.1	How mentally demanding was the task? (0–100)
Q4.2	How physically demanding was the task? (0–100)
Q4.3	How hurried or rushed was the pace of the task? (0–100)
Q4.4	How successful were you in accomplishing what you were asked
	to do? (0–100)
Q4.5	How hard did you have to work to accomplish your level of
	performance? (0–100)
Q4.6	How insecure, discouraged, irritated, stressed, and annoyed were
	you? (0–100)
Q5	What comments do you have about your experience performing
	this construction session? (text)

	Post-trial questions
Q6	What differences did you notice, if any, between three construction
	sessions? (text)
Q7	Which construction trial was the fastest to complete? (1, 2, or 3)
Q8	Which construction trial was the easiest to complete? (1, 2, or 3)
Q9	Which construction trial was the most fatiguing? (1, 2, or 3)
Q10	Which construction trial did you like best? Why? (1, 2, or 3; text)
Q11	For the two sessions that included this step, how did you decide
	what gain level to choose? (text)
Q12	Do you have any additional comments about the study? (text)

For each assembly trial, the participant had 5 minutes to become familiar with the system settings and tasks (practice section), and then they were asked to complete the three-step assembly task within 20 min (experimental section). During the practice section, the participant was allowed and encouraged to adjust the system by tuning the vibrotactile feedback gain according to their preference from a minimum of −20 dB to a maximum of 10 dB. The gain chosen by the participant was recorded and kept constant for the rest of the trial.

The participant repeated this 25-minute-long assembly trial (including practice and experimental sections) under the three different haptic feedback conditions (F0, F1, F3). Following each trial, the participant completed the post-trial questionnaire. To control for order effects and learning, we randomly assigned the thirty participants to six groups; each group experienced the conditions in one of the six possible presentation orders.

### 3.3 Sample trial data

**FIGURE 6:**
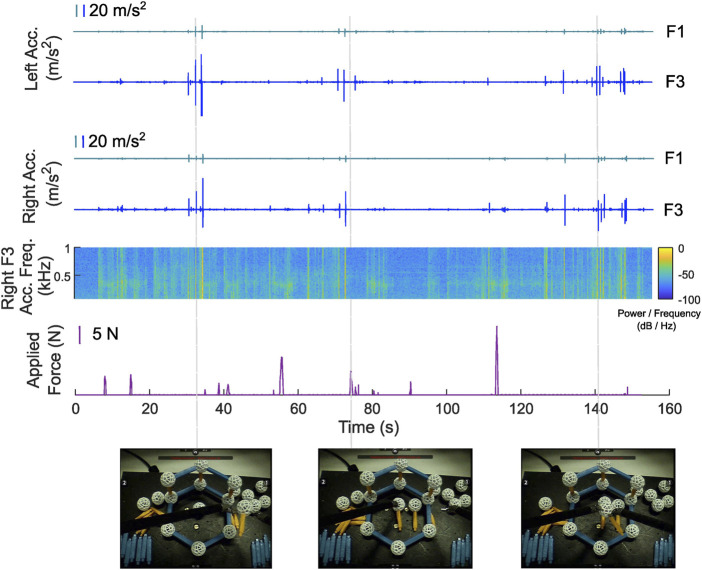
Tool vibrations and applied force magnitude for step 1 of one trial in the study. Each tool acceleration shows both the F1 and F3 signals; as F3 is the sum of the three signals sensed by the accelerometer, it has larger peaks than F1. The acceleration spectrogram shows the instantaneous frequency content of the F3 signal of the right tool. The screenshots at the bottom show the participant’s left eye view of the task materials at the three indicated time points. The first and third screenshots show the insertion of a stick into a sphere in the air, where only an acceleration transient was generated. The second screenshot shows the placement of an assembled piece on the baseplate, where mainly an applied force was detected without an acceleration transient.

Figure 6 shows sample acceleration and force data from one participant performing step 1 of the assembly task. We depict both the F1 and F3 signals for both tools, as well as three screenshots of the participant’s left eye view for context. It is important to note that the summed three-axis feedback accelerations (F3) have larger peaks than the one-axis F1 acceleration signals. The supplementary video associated with this article shows this same interaction along with animated plots of the corresponding left tool vibration, right tool vibration, and applied force magnitude over time.

### 3.4 Evaluation metrics and statistical analysis

The system noise was recorded for 1 minute without moving the robot to identify a noise threshold that could be used for data analysis after the study. The threshold was set to 1.05 times the maximum absolute value of the noise measured during this time for both the acceleration (0.3 m/s^2^) and the force vector magnitude (0.2 N). Acceleration and force measurements with a magnitude above the threshold were retained, and the data below were set to 0 for analysis. We confirmed that the noise level was consistent across all datasets.

The RMS and zero-crossing-rate (ZCR, the number of times per second the signal crosses zero) of the thresholded acceleration and force signals were used to quantify the signal’s strength and the average interaction intensity, respectively. We also evaluated the completion level of each trial based on the number of sticks successfully assembled by the operator. Each end of a stick was counted as 0.5. Thus, the completion level necessary for both step 1 and step 2 was 1.5, and it was 4 for step 3, totaling a maximum completion level of 7 for each trial.

Due to the relatively low number of participants and the fact that the data are not normally distributed, we performed non-parametric statistical analyses. The Spearman correlation was calculated on the combined data from all three trials for all participants (*N* = 90 trials) to evaluate how the tool acceleration metrics relate to the force metrics, and how each of these metrics is associated with the mean HR, completion time, hand volume, TLX workload, and trial number. Correlations were also used to examine the relationship between the user hand volume, the haptic feedback condition, and the chosen feedback gain, as well as the association between the trial number and all the metrics.

We used Friedman tests (within subjects, *N* = 90) to investigate the general effects of the three feedback conditions on the acceleration and force metrics, completion time, mean HR, and the post-trial questionnaire scores. When we obtained significant results, we then used the Wilcoxon signed-rank tests for *post hoc* analysis on every pair of the three conditions. Wilcoxon signed-rank tests were also used for the gain selection analysis.

As explained in Section 3.2, participants were assigned to one of the three haptic feedback conditions in each trial. Kruskal-Wallis tests were used to analyze the effect of the feedback conditions in each trial separately (between subjects, *N* = 30); we employed the Mann-Whitney test for the *post hoc* analysis on every pair of conditions within each trial.

We used *α* = 0.05 to determine statistical significance, and the Bonferroni-Holm correction was applied to correct the significance levels for multiple comparisons.

## 4 Results

We used the accelerometers on the cannulas to measure the magnitude of the tool vibrations, the force sensor to measure the applied force, the task completion time and completion level to evaluate the assembly efficiency, the heart rate (HR) tracker to evaluate the operator’s mental stress, the chosen haptic feedback gains to check the effects of the two versions of haptic feedback, and the questionnaire responses to evaluate workload and overall system performance.

### 4.1 Task completion level

All thirty participants finished the first two steps of the experiment in every trial, but some of them did not finish the third step within the time limit. In trial 1, 11 participants did not complete the third step; among them, one participant achieved a completion level of 0.5 out of 4, one got 1.5, four earned 2, and five achieved 3. The number of incomplete structures in step 3 decreased to five in trial 2; here, three participants reached a completion level of 2, while two participants earned 3. Only one participant failed to build the entire structure in trial 3, with a completion level of 2. Participants clearly gained experience over the course of the experiment.

### 4.2 Correlations between metrics

When two metrics are positively correlated, one increases as the other increases; a negative correlation means one metric increases while the other decreases. We found all metrics for acceleration and force are positively correlated with one another (*r*_*s*_ > 0.260, *p* < 0.015 for all), except for the correlations between force RMS and acceleration ZCR for each tool.

The acceleration RMSs of both tools have positive correlations with mean HR (Left: *r*_*s*_ = 0.427, *p* < 0.001; Right: *r*_*s*_ = 0.369, *p* < 0.001) and negative correlations with completion time (Left: *r*_*s*_ = −0.314, *p* = 0.003; Right: *r*_*s*_ = −0.290, *p* = 0.006). The acceleration ZCRs of both tools have positive correlations with mean HR (Left: *r*_*s*_ = 0.472, *p* < 0.001; Right: *r*_*s*_ = 0.533, *p* < 0.001), and they have negative correlations with completion time (Left: *r*_*s*_ = −0.360, *p* < 0.001; Right: *r*_*s*_ = −0.331, *p* = 0.001). The acceleration ZCR of the left tool is positively correlated with the TLX workload (*r*_*s*_ = −0.259, *p* = 0.014). The force ZCR also has a positive correlation with mean HR (*r*_*s*_ = 0.371, *p* < 0.001) and a negative correlation with completion time (*r*_*s*_ = −0.245, *p* = 0.02).

The trial number is negatively correlated to completion time (*r*_*s*_ = −0.425, *p* < 0.001) and mean HR (*r*_*s*_ = −0.232, *p* = 0.028). The TLX workload is positively correlated with completion time (*r*_*s*_ = −0.263, *p* = 0.012). No significant correlations were found in the rest of the metric pairs.

### 4.3 The effect of haptic feedback

#### 4.3.1 General effect of the three feedback conditions

We found a significant main effect of feedback condition only on the right tool acceleration RMS (*χ* ^2^ (2) = 8.600, *p* = 0.014). No significant differences were found in any other metrics. Post-hoc analysis shows F0 has a significantly higher RMS acceleration than F1 (*Z* = −2.499, *p* = 0.036, ${\bar{AR}}_{F0}=0.454$ m/s^2^, ${\bar{AR}}_{F1}=0.418$ m/s^2^), and F0 also tends to be higher than F3 (*Z* = −2.232, *p* = 0.052, ${\bar{AR}}_{F3}=0.415$ m/s^2^). No significant difference was found between F1 and F3.

For the post-trial questionnaires, there is a significant main effect of feedback condition in realistic interaction (*χ* ^2^ (2) = 14.460, *p* < 0.001), and F1 and F3 are significantly more realistic than F0 in *post hoc* analysis (F0 vs. F1: *Z* = −3.528, *p* < 0.001; F0 vs. F3: *Z* = −3.618, *p* = 0.004; ${\bar{R}}_{F0}=46.567$ , ${\bar{R}}_{F1}=64.733$ , ${\bar{R}}_{F3}=63.000$ ). No significant differences were found in any other questions.

#### 4.3.2 Effect of the three feedback conditions within each trial

**FIGURE 7:**
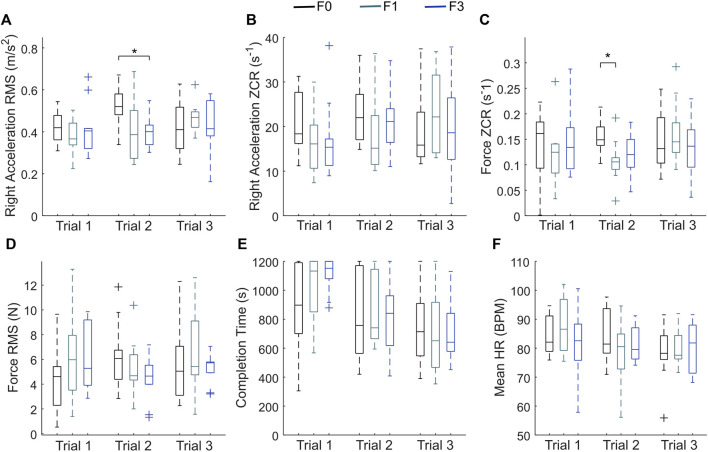
The accelerometer and force data analyzed by trial. **(A)** RMS of summed acceleration from the right accelerometer (dominant hand); **(B)** ZCR of right acceleration; **(C)** ZCR of force; **(D)** RMS of force; **(E)** Completion time; **(F)** Mean HR. The bottom and top of the box represent the 25% and 75% responses, and the center line is the median. The + marks indicate outliers. The lines extending past the boxes show the farthest data points not considered outliers. Statistically significant pairwise differences are marked with*.

Since the task was repeated three times, participants naturally gained experience over time. Indeed, as indicated by the correlation results, we observed a significant association between the trial number and the completion time, as well as the mean HR, despite the feedback randomization. To further control for the potential impact of the trial number on the effects of haptic feedback, we also analyzed the metrics for each trial individually. Figure 7 shows the effect of feedback condition on the right tool accelerations and force variables by trial.

In trial 1, there is a significant main effect of haptic feedback on completion time (*χ* ^2^ (2) = 6.115, *p* = 0.047). Post-hoc analysis indicates that the completion time of F0 has a trend to be lower than that of F3 (*U* = 16.500, *p* = 0.051, ${\bar{T}}_{F0}=867.200$ s, ${\bar{T}}_{F3}=1107.900$ s). No significant differences were found in other metrics in trial 1. In trial 2, there is a significant main effect of feedback condition on the right tool RMS acceleration (*χ* ^2^ (2) = 7.208, *p* = 0.027) and force ZCR (*χ* ^2^ (2) = 6.978, *p* = 0.003). Post-hoc analysis shows the right tool acceleration RMS of F0 is significantly higher than that of F3 (*U* = 17.000, *p* = 0.033; ${\bar{AR}}_{F0}=0.525$ m/s^2^, ${\bar{AR}}_{F3}=0.402$ m/s^2^), and the force ZCR of F0 is significantly higher than that of F1 (*U* = 16.000, *p* = 0.03; ${\bar{FZ}}_{F0}=0.155$ s^−1^, ${\bar{FZ}}_{F1}=0.122$ s^−1^). No other significant differences were found in trial 2. In trial 3, no significant differences were found in any signal-based metrics.

No significant differences were found in the scores of the post-trial questionnaires in trial 1 and 2. However, in trial 3, F1 and F3 both trend to give users more confidence in moving the parts (*χ* ^2^ (2) = 5.879, *p* = 0.053) and more realistic interaction with the building materials (*χ* ^2^ (2) = 5.502, *p* = 0.064) compared to F0.

#### 4.3.3 Gain selection

There were two outliers in the gain choices, −20 dB in F1 and −16 dB in F3, where two participants opted not to have the haptic feedback when they were supposed to. One of these participants mentioned they relied more on vision, leading to a preference for a very weak gain. The other participant mistakenly selected a weak gain in the first trial and could not feel the feedback, so they greatly increased the gain level in the subsequent trial. Therefore, we removed the data for these two participants for the gain selection analysis. We converted the chosen gains from decibels to amplitude to achieve linearity with actuator force, and we averaged the gain choices for each remaining participant (N = 28). The average gain is positively correlated with the measured hand volume (*r*_*s*_ = 0.474, *p* = 0.01). We also compared the gains participants chose for F1 and F3. On average, the gain for one-axis feedback is significantly higher than for summed three-axis feedback (*Z* = −2.959, *p* = 0.003, F1: 1.92 ± 0.95; F3: 1.25 ± 0.72).

#### 4.3.4 Post-experiment questionnaire

**FIGURE 8:**
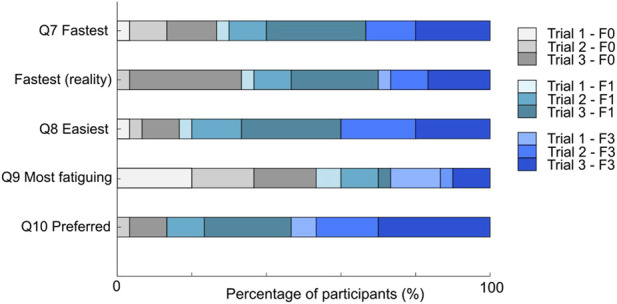
The results of Q7 to Q10. The participant was asked to identify the fastest trial, the easiest trial, the most fatiguing trial and the trial they like the most. The second graph shows the real fastest trial calculated from the recorded completion time.

84% of the participants said they could tell the difference between with and without haptic feedback in Q6, while only 6% of them mentioned that the two haptic feedback conditions felt different. We compared the participants’ responses for Q7 to Q10 with their assigned feedback conditions. The results of these four questions are shown in Figure 8. 77% of the participants chose a trial with haptic feedback (F1 or F3) as the fastest to complete. Surprisingly, based on the measured completion time, almost half (47%) of the participants did not choose their actual fastest trial as the fastest completed trial. Trial 3 with no haptic feedback had the highest number of unmatched answers. For easiest trial, 84% of the participants chose a trial with haptic feedback. For most fatiguing trial, only 54% chose a trial with haptic feedback. For favorite trial, 87% of the participants chose one with haptic feedback. Their reasons were diverse, such as the feedback helps provide a more realistic experience, it is helpful for beginners to understand the assembly strategies, and it helps when visual feedback is insufficient. Several participants also commented that haptics is usually taken for granted and undervalued during their daily lives.

For the open-ended Q11 about gain selection, 50% of the participants said they chose the gain to be neither too strong nor too weak, and 17% of the participants said they set the gain to be at a comfortable level. 23% of the participants said they changed their preference during the experiment, as they preferred strong vibration initially, and then they realized that somewhat weaker vibration is better and turned it down in the subsequent trial. In the free comments, 10% of the participants said they did not find the vibrotactile feedback to be helpful, because they found it distracting or strongly relied on visual feedback. One of the participants mentioned that they did not realize the importance of haptic feedback until they experienced conditions with and without it.

## 5 Discussion

The measurements presented in Section 2 strongly support ***H1*** and were used to select higher-performance locations for mounting AiroTouch sensors and actuators to a da Vinci Si teleoperation system. Overall, the results from the subsequent user study strongly support our hypotheses that naturalistic haptic feedback of tool vibrations has positive effects on the performance of assembly activities in both objective (***H2***) and subjective (***H3***) evaluations. However, the highly similar results found for the one-axis and summed three-axis feedback do not support ***H4***. Finally, the gain choice is positively correlated to the hand size and negatively correlated to the intensity of the vibrotactile signal being presented, as predicted by ***H5***.

### 5.1 Objective evaluation

During assembly activities, strong contact vibrations and forces would deform or damage the structure being built; buildings with smaller construction-induced deformations last longer (Atkinson, 2003). Thus, we regard it as better performance when participants assemble the components with smaller generated tool vibrations and baseplate forces.

The objective analysis of our study supports ***H2***. Providing naturalistic haptic feedback of tool vibrations generally reduced the vibrations and forces that the user caused while assembling the structure. The acceleration RMS of the right tool, which was controlled by the participant’s dominant hand, was significantly lower when either type of haptic feedback was provided (Section 4.3). After participants became familiar with the setup during the first trial, they caused lower vibrations and lower forces when they received haptic feedback. Specifically, we observed statistically significant lower values for two calculated metrics in trial 2: right-tool acceleration RMS in F3 compared to F0 and force ZCR in F1 compared to F0 (Figure 7). The positive correlation between tool accelerations and contact forces presented in Section 4.2 reinforces this connection. These results provide the first evidence that naturalistic vibrotactile feedback without force feedback improves the quality of the teleoperated robot’s interactions with the materials being assembled, reducing both tool accelerations and exerted forces.

We did not observe any significant effect of haptic feedback in the four robot-sensor-based metrics (RMS and ZCR for accelerations and forces) in trial 1 or trial 3. In trial 1, regardless of the feedback condition, users tended to be more cautious, as it was their first time being exposed to the robot and task. The participants who did not have haptic feedback commented that they had no concept of the robotic tool strength, and they sometimes damaged the toy structure. In contrast, the participants who received haptic feedback at the beginning said they had a more realistic experience and were motivated to complete the task. Based on their comments, it was evident that the participant could tell the robot’s strength from the magnitude of the vibration feedback during the task. This knowledge enabled them to adapt their movements to minimize potential damage during trial 2. In trial 3, the metric values were similar in all feedback conditions. The participants commented that naturalistic vibrotactile feedback would be particularly beneficial for beginners during the initial stages when they are exploring assembly strategies. As they gained more experience with the teleoperation system and the task, their performance improved through learning, regardless of their current feedback condition. Several users mentioned that they relied on the experience gained from previous trials, hinting that the benefits of past exposure to naturalistic vibrotactile feedback may persist when the feedback is removed. Longer studies with only one change in feedback type are needed to investigate this idea.

We found that hand size was positively correlated to a participant’s average gain choice, supporting the first half of ***H5***, that individuals with larger hands will generally prefer stronger vibrotactile actuation. Additionally, we observed that participants tended to choose higher gains for the smaller vibration feedback signals provided in F1, supporting the second half of ***H5***. Both of these results indicate that users generally prefer to amplify the sensations of typical physical collisions to approximately the same vibrotactile magnitude at their fingertips, potentially trying to match the vibrotactile transients felt during direct tool use. However, despite these two significant findings, large variations in gain preferences were seen across individuals, most likely due to differing tactile sensitivity and personal preferences.

### 5.2 Subjective evaluation

Mental health is crucial in construction. Due to the dangerous working environment and the high workload, construction workers often feel stress and anxiety (Abdelhamid and Everett, 2002), which may increase accident rates (Leung et al., 2010; Langdon and Sawang, 2018). We envision that naturalistic feedback of the vibrations experienced by teleoperated construction robots could help create a lower-pressure and therefore safer environment for workers by making assembly tasks easier to perform.

The questionnaire responses support ***H3*** about the subjective benefits of this type of haptic feedback. As found for surgical training tasks with VerroTouch (McMahan et al., 2011; Koehn and Kuchenbecker, 2014), most participants preferred having naturalistic vibrotactile feedback available when doing assembly activities. We attribute this preference to the fact that most participants thought they had more realistic interactions with the building materials and that they finished the task faster, more easily, and with less fatigue when they had haptic feedback, as described in Section 4.3. The result of the fastest trial (Q7) is mildly surprising: A group of participants thought they finished the task most quickly when they had haptic feedback, rather than realizing that their final trial without haptic feedback was actually the fastest. Thus, we anticipate that this form of haptic feedback may help build their confidence and create a more immersive work environment. Another supportive result is that more than two-thirds of participants thought the trials with haptic feedback were easier (Q8). Our results about fatigue (Q9) also show the positive mental effects of haptic feedback. Furthermore, as described in Section 4.2, the calming effect of haptic feedback is also supported by the positive correlation between mean HR and both acceleration RMS and ZCR, showing haptic feedback tends to reduce tool vibrations, and users had lower heart rate on trials with lower tool vibrations. Even though we did not detect any significant differences in the TLX results, the questionnaire responses provided valuable insights into the participants’ perspectives.

We hypothesized that participants would be more confident (less stressed) when receiving haptic feedback and thus have a lower mean HR in those conditions; however, our analysis did not reveal any significant effects of feedback on mean HR beyond the mentioned correlations. Anecdotally, participants without haptic feedback in the first trial tended to have a higher heart rate, as did participants who caused the items being assembled to fly away from the platform by applying high forces, which happened most often without haptic feedback. Our finding of no differences in mean HR across feedback conditions may be due to the relatively short trial duration, insufficient breaks between conditions (needed to return to resting HR), the low sampling rate of the selected heart rate tracker, and the fact that we did not collect a baseline HR.

The naturalistic vibrotactile feedback provided by AiroTouch showed the participant the intensity of the contacts they were making with the task materials. Participants who started with haptic feedback immediately began making more conservative movements, which caused them to complete the task more slowly in this first trial (Section 4.3). From this perspective, it is possible that naturalistic vibrotactile feedback both increases the operator’s stress in the short term, by giving them more information to process and try to optimize for, while also reducing their stress over a longer term, by enabling higher-quality manipulation with lower forces and vibrations. This connection merits further investigation.

Interestingly, we did not observe any differences between F1 and F3 from the questionnaires, nor did we find any consistent differences between F1 and F3 in the objective metrics (Figure 7), indicating that our hypothesis ***H4*** is not supported. The majority of the subjects could discern only whether there was haptic feedback, and they could not differentiate between the two types; indeed, the F1 and F3 feedback signals measured in this study were quite similar to one another after amplification. Only a few participants commented that they felt F1 was cleaner, or that F3 was more informative. Thus, we can conclude that people could not distinguish between one-axis feedback and summed three-axis feedback in this study. More investigation would be needed to determine whether a preference or performance difference would exist for other robots and tasks. Other studies also need to be conducted to compare naturalistic vibrotactile feedback to other types of haptic, visual, and auditory feedback that could be deployed in such scenarios, both alone and in combination with one another.

## 6 Conclusion

Motivated by the difficult task of assembling prefabricated building components on construction sites, this paper presented a reliable haptic feedback system that uses off-the-shelf equipment to provide users with naturalistic real-time vibrotactile feedback while observing or teleoperating a robot. The system consists of high-bandwidth accelerometers, an audio mixer, audio amplifiers, and voice-coil actuators; one accelerometer, audio amplifier, and actuator are needed for each robotic tool. We found that adjusting the placement of the sensors and actuators can improve the quality of the resulting vibrotactile feedback, supporting ***H1***. Then we conducted a user study to evaluate this system and explore the effects of naturalistic vibrotactile feedback during telerobotic assembly. Thirty participants used a da Vinci robot to assemble a structure in three randomly ordered haptic feedback conditions: no feedback, one-axis feedback, and summed three-axis feedback. Both types of haptic feedback reduced the vibrations of the right tool across the study. More specifically, participants receiving either form of haptic feedback produced fewer force oscillations and smaller tool vibrations in trial 2, supporting ***H2***. Most participants showed a strong preference for haptic feedback in their qualitative evaluations, believing that it makes the task easier and less fatiguing, supporting ***H3***. Interestingly, our participants could not distinguish between one-axis and summed three-axis feedback, undermining ***H4***. Participants with larger hands generally chose higher feedback gains, and higher gains were also chosen for the smaller measured vibration signals of the one-axis feedback, supporting both parts of ***H5***.

This paper’s findings about the objective and subjective benefits of naturalistic vibrotactile feedback support its use on teleoperated robots in construction as well as other applications such as surgery, search and rescue, and hazardous material handling. The presented system design and sensor and actuator placement strategies aim to simplify adoption by a wide range of stakeholders in these fields, from other researchers to companies and even end-users.

Several limitations should be considered when interpreting this study’s results. Due to safety concerns and the low accessibility of construction robots for our research, we used a small-scale surgical teleoperation robot to evaluate our haptic feedback system, which could raise concerns regarding scale and application domain. In addition, we mainly processed the measured accelerations as audio signals. A signal propagation model could be built to support selection of the optimal locations for the sensors and actuators by analyzing the structural resonances and harmonic frequencies. Moreover, we did not compare the performance of naturalistic vibrotactile feedback with other kinds of haptic feedback; such a comparison should be done in the future. Our own interests still center on leveraging beneficial digital technologies to advance construction, due to the major societal, economic, and environmental impacts of this sector (Melenbrink et al., 2020). Thus, our future work will implement AiroTouch for the operator of a real construction robot performing more realistic tasks in a large workspace with appropriate safeguards to preserve human, machine, and material safety.

## Data Availability

The raw data supporting the conclusion of this article will be made available by the authors, without undue reservation.
